# Cell signaling in *Ehrlichia* infection and cancer: Parallels in pathogenesis

**DOI:** 10.3389/fcimb.2025.1539847

**Published:** 2025-02-14

**Authors:** Regina N. Solomon, Nicholas A. Pittner, Jaclyn R. McCoy, Paityn A. Warwick, Jere W. McBride

**Affiliations:** ^1^ Department of Pathology, University of Texas Medical Branch, Galveston, TX, United States; ^2^ Department of Microbiology and Immunology, University of Texas Medical Branch, Galveston, TX, United States; ^3^ Center for Biodefense and Emerging Infectious Diseases, University of Texas Medical Branch, Galveston, TX, United States; ^4^ Sealy Institute for Vaccine Sciences, University of Texas Medical Branch, Galveston, TX, United States; ^5^ Institute for Human Infections and Immunity, University of Texas Medical Branch, Galveston, TX, United States

**Keywords:** *Ehrlichia*, apoptosis, cancer, short linear motif, notch, Wnt, hedgehog, hippo

## Abstract

*Ehrlichia chaffeensis* (*E. chaffeensis*) has recently emerged as an intracellular bacterial pathogen with sophisticated survival mechanisms that include repurposing evolutionarily conserved eukaryotic cell signaling pathways for immune evasion. *E. chaffeensis* exploits four major developmental signaling pathways (Wnt, Notch, Hedgehog, and Hippo) using short linear motif (SLiM) ligand mimicry to initiate signaling cascades. Dysregulation of these major signaling pathways leading to unchecked cell survival is implicated in various diseases, most notably cancer. *E. chaffeensis* exploits Wnt, Notch, Hedgehog and Hippo signaling pathways to inhibit apoptosis and co-opt other cellular functions to promote infection. This review will explore the signaling pathways exploited during *Ehrlichia* infection and the new discoveries that have illuminated this interesting example of the cell signaling convergence in cellular infection and cancer biology.

## Introduction

**Figure 1:**
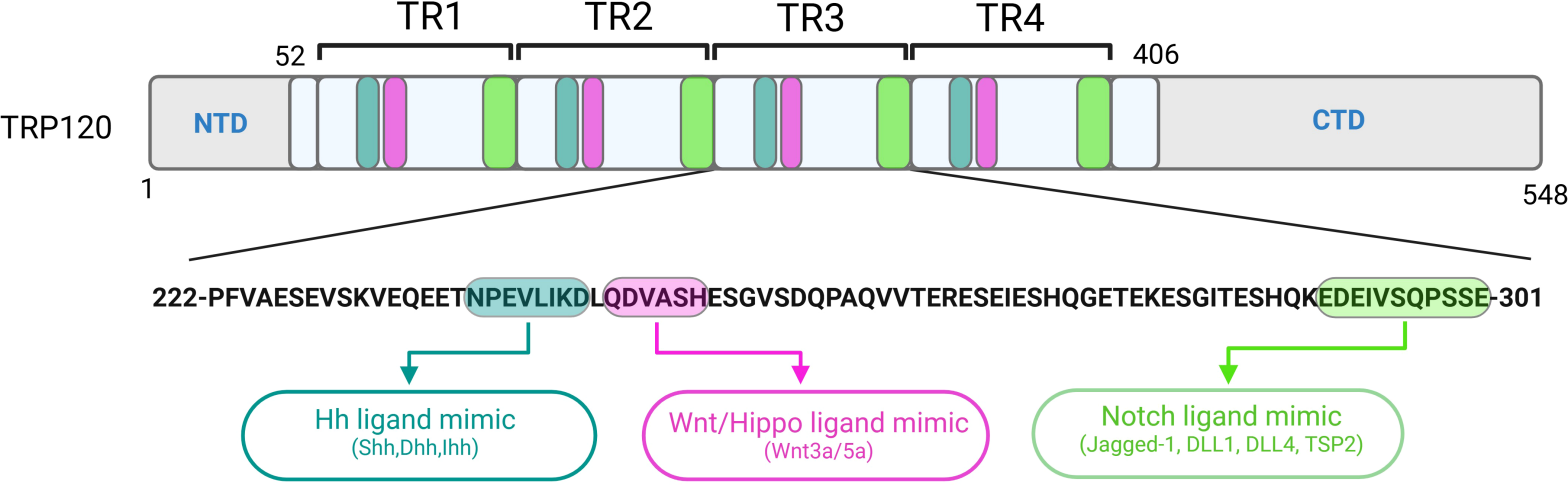
Localization of functionally characterized *E. chaffeensis* TRP120 SLiMs. Repetitive Hh, Wnt, Hippo and Notch SLiMs located within each tandem repeat (TR) domain of TRP120 have been experimentally validated and shown to activate respective pathways during infection.

*Ehrlichia chaffeensis* is a gram-negative, obligately intracellular rickettsial pathogen and the etiologic agent of human monocytotropic ehrlichiosis (HME), an emerging life-threatening tick-borne zoonosis of increasing public health importance (Paddock and Childs, 2003). *E. chaffeensis* preferentially replicates in mononuclear phagocytes by effectively reprogramming the host cell through secreted tandem repeat effectors, most notably the 120 kDa tandem repeat protein (TRP120). Over the past decade, TRP120 has become recognized as a multifunctional “moonlighting” effector acting as a transcription factor, invasin, HECT E3 ubiquitin ligase, and most remarkably, a ligand mimic for multiple signaling pathways (**Figure 1**) (Zhu et al., 2011; Wang et al., 2020; Pittner et al., 2023). In fact, TRP120 is the first bacterial effector described capable of complex multi-pathway ligand mimicry driven by short linear motifs (SLiMs). SLiMs are small, functionally diverse protein interaction modules involved in regulatory interactions within the cell (Van Roey et al., 2014). While classical protein-protein interactions often depend on complex tertiary structures, recent advances have shown that interactions also occur via SLiM-globular and intrinsically disordered domain (IDD)-globular interfaces (Van Roey et al., 2014).

Over the past two decades, structural studies have revealed that a significant proportion of eukaryotic proteins are intrinsically disordered (Tompa, 2011, 2012). While they lack a well-defined tertiary structure, intrinsically disordered regions exhibit various cellular functions encoded within short sequences, now recognized as SLiMs (Davey et al., 2012). SLiMs have been identified as an *ex-nihilo* evolutionary adaptation, introducing functional interfaces to previously non-functional regions of proteins (Davey et al., 2015). Driving protein-protein interactions, SLiMs have been shown to regulate immune and inflammatory responses, cell proliferation, differentiation, and apoptosis, as well as control protein stability, recruit substrates, direct cellular trafficking and pose as sites for post-translational modifications (PTMs) and proteolytic cleavage (Davey et al., 2015; Sologova et al., 2022). The recognition of SLiMs has reshaped our understanding of cell biology with insurmountable evidence demonstrating SLiMs are reframing the paradigms of cellular regulation through eukaryotic protein interactions as well as pathogen-host interactions during infection (Neduva and Russell, 2005; Stein and Aloy, 2008; Van Roey et al., 2014).

The Eukaryotic Linear Motif (ELM) database is a computational biology resource with an expanding catalog of 4,277 experimentally validated SLiMs. According to an ELM prediction, *E. chaffeensis* TRP120 has 45 unique SLiM classes and 189 total SLiM instances (Kumar et al., 2024). This SLiM inventory does not include the recently described Wnt, Notch, Hedgehog (Hh) and Hippo mimics, suggesting that many TRP120 SLiMs remain to be identified and that most of the predicted TRP120 SLiMs have not been functionally validated. To date, the most definitive studies of *E. chaffeensis* TRP120 SLiM function are related to PTMs that affect pathogen-host interactions and those involved in cell signaling which impacts bacterial entry and innate immune defenses including autophagy and TLR expression, and perhaps most importantly apoptosis, to create a suitable niche for intracellular survival (Lina et al., 2016, 2017; Luo et al., 2016; Wang et al., 2020; Patterson et al., 2023). In the context of *Ehrlichia* infection, SLiM ligand mimicry (SLiM-icry) is used to engage host cell receptors and activate evolutionarily conserved signaling pathways (Pittner et al., 2023). Multiple instances of unique SLiM-icry are present in the tandem repeat domain of TRP120, capable of directly activating/deactivating Notch, Wnt, Hh and Hippo signaling pathways during ehrlichial infection (**Figure 1**) (Pittner et al., 2023). The Wnt pathway was the first evolutionarily conserved signaling pathway shown to be co-opted by *E. chaffeensis* SLiM-icry (Rogan et al., 2021). The revelation of this sophisticated survival strategy led to the discovery that *E. chaffeensis* also uses SLiM-icry to exploit Notch, Hh and Hippo (Byerly et al., 2022, 2023; Patterson et al., 2022).

Considering what is known about Wnt, Notch, Hh, and Hippo signaling pathways, the intersection between immune evasion strategies employed by *E. chaffeensis* and the hallmarks of cancer biology is intriguing. Dysfunction of these pathways has been appreciated in cancer biology as well as in other developmental diseases for decades (Kling and Blumenthal, 2017; Zheng and Pan, 2019; Kumar et al., 2021; Fu et al., 2022; Sharma et al., 2022), however, understanding the dysregulation of evolutionarily conserved cellular signaling pathways in cancer biology and its resistance to anticancer therapies is a major hurdle for improving therapeutic approaches to cancer treatment (Liang et al., 1999; Hanahan and Weinberg, 2011; Kumar et al., 2021). Interestingly, there are similarities in the molecular survival strategies employed by *E. chaffeensis* and the oncogenic mechanisms in malignant cancer cells that may provide synergistic insight to both areas of research. This is further supported by various TRP120 SLiMs identified by the ELM database that are implicated in carcinogenesis such as Src homology 2 (SH2) interaction motif (LIG_SH2_STAT3), BRCA1 tumor suppressor binding domain (LIG_BRCT_BRCA1_1), retinoblastoma protein interaction motif (LIG_RB_LxCxE_1) and MAP kinase docking site (DOC_MAPK_MEF2A_6) (Pittner et al., 2023; Kumar et al., 2024). While numerous pathogens utilize similar mechanisms to reprogram host cellular pathways (Holla et al., 2016; Konstantinou et al., 2016; Smelkinson, 2017; Smelkinson et al., 2017; Iriana et al., 2021), *E. chaffeensis* has emerged as a model pathogen adept at modulating molecular mechanisms involved in signaling pathway activation and regulation that may be useful in understanding cancer biology. This review will summarize key cellular and molecular insights and implications for advancing our knowledge of *E. chaffeensis* immune evasion as well as cancer biology.

## Wnt pathway

Wnt signaling is an evolutionarily conserved pathway first discovered in 1982 as proto-oncogene “Int-1” in mice and was later revealed as the homolog of the “wingless” gene in *Drosophila*. Wnt pathway components are comprised of more than sixteen, mammalian, cysteine-rich secreted ligands necessary for canonical and noncanonical Wnt pathway activation. Wnt pathway activation is initiated when Wnt ligands bind the extracellular domain of Frizzled (Fzd) receptors which dimerize with coreceptors lipoprotein receptor-related protein (Lrp) 5, -6, or tyrosine kinase-like orphan receptor (Ror) 2 to subsequently activate disheveled (Dvl). Canonical Wnt is defined as β-catenin-dependent, whereas noncanonical Wnt is β-catenin-independent. Two distinct noncanonical pathways have been described: Calcium (Ca^2+^) and Planar Cell Polarity (PCP). Canonical Wnt/β-catenin signaling controls cellular proliferation and differentiation, and is important in embryogenesis, organogenesis, and homeostasis. Conversely, activation of non-canonical Wnt pathways primarily results in regulation of cell motility and polarity (Di Bartolomeo et al., 2023). Wnt signaling is essential for embryonic development, cellular differentiation, polarization, as well as the control and growth of stem cells. Therefore, it is unsurprising that aberrant signaling has been implicated in various diseases including neurodegenerative, metabolic, and inflammatory diseases, as well as various cancers (Duchartre et al., 2016). Furthermore, Wnt signaling is involved in regulation of innate immune responses making it an important target for infectious agents (Silva-García et al., 2014; Jati et al., 2019; Mukherjee and Balaji, 2019; Rogan et al., 2019).

### Wnt signaling in cancer

Wnt signaling has been linked to various types of cancers including colon, cutaneous melanoma, hepatocellular carcinoma (HCC) and breast cancer. It is also involved in metastasis as Wnt regulates cell morphology and motility. Increased Wnt ligand 5a (Wnt5a) in melanoma was correlated with increased invasiveness, cell motility and changes in morphology through changes in calcium signaling. Wnt5a has been extensively associated with proto-oncogenic cellular phenotypes. Wnt5a has been shown to act as a proto-oncogene in melanoma, breast cancer, prostate and pancreatic cancer, and a tumor suppressor in breast cancer, colon, thyroid and esophageal squamous cell carcinoma, acute lymphoblastic lymphoma, acute myeloid lymphoma, and neuroblastoma (Taciak et al., 2018).

In canonical Wnt signaling, binding of nuclear β-catenin to TCF/LEF transcription factors stimulate expression of cyclin D1 and c-MYC which alters cell cycle progression and promotes tumorigenesis in cutaneous melanoma (Taciak et al., 2018). β-catenin/TCF2 is a negative regulator of IFIT1 and IFIT2, host antiviral defense mediators through apoptosis. In colorectal cancer, IFIT2 expression is decreased which creates a pro-survival environment for cancer cells through inhibition of apoptosis. The β-catenin/TCF2 complex and down regulation of IFIT1/2 is commonly seen in colorectal cancer compared to normal tissue (**Table 1**) (Taciak et al., 2018).

**Table 1:** Parallels in cell signaling across *Ehrlichia* infection and cancer.

Pathway	Protein	Function in *Ehrlichia*	Function in cancer	References
Wnt	FZD5	TRP120 SLiM-activated receptor of Wnt and Hippo signaling during infection	Initiates Wnt signaling in triple negative breast cancer (TNBC) to promote DNA damage repair and enhance chemoresistance.	(Sun et al., 2020; Rogan et al., 2021; Byerly et al., 2023)
	β-catenin	Canonical Wnt transcription factor, increased nuclear localization during infection associated with enhanced bacterial entry.	Inhibits apoptosis in colorectal cancer cells via negative regulation of host defense mediators, (IFIT1/2).	(Luo et al., 2016; Taciak et al., 2018; Rogan et al., 2021)
	NFAT	Non-canonical Wnt transcription factor activated during infection; function unknown.	Downstream target of α4β6 integrin signaling enhancing cell migration and carcinoma invasion.	(Jauliac et al., 2002; Luo et al., 2016)
Notch	Notch1	TRP120 SLiM-activated receptor of Notch signaling during infection.	Promotes proliferation and inhibits apoptosis in pancreatic cancer via APOL-mediated activation.	(Lin et al., 2021; Patterson et al., 2022)
	NICD	Canonical Notch transcription factor, binds and sequesters XIAP and shown to delay host cell apoptosis.	Initiates lung tumorigenesis in conjunction with MYC activity in knock-in mouse models.	(Allen et al., 2011; Patterson et al., 2022, 2023)
	ADAM17	Metalloprotease associated with Notch activation, shown to interact with TRP120 during infection.	Promotes EMT transition via TGF-β/Smad pathway contributing to gastric cancer progression.	(Lina et al., 2016; Ni et al., 2020; Patterson et al., 2022)
	XIAP	Interaction with NICD stabilizes expression and promotes delayed host cell apoptosis during infection.	Downregulation via small molecule inhibitor decreased cell viability and induced apoptosis in breast cancer cells.	(Hussain et al., 2017; Patterson et al., 2023)
	FBW7	TRP120-mediated degradation leads to upregulation of oncoproteins (c-MYC, NICD,c-Jun and MCL1) during infection.	Decreased expression via ERK-mediated degradation disrupts tumor-suppressive activity in pancreatic cancer.	(Ji et al., 2015; Wang et al., 2020)
	PU.1	Transcription factor responsible for TLR2/4 expression, downregulated during infection to disrupt immune response.	Exhibits tumor-suppressive effects by inhibiting cell migration and promoting apoptosis via BCL-2 inhibition, downregulated in lung adenocarcinoma tissues.	(Lina et al., 2016; Liu et al., 2023b)
Hedgehog	PTCH2	TRP120 SLiM-activated receptor of Hh signaling during infection	Enhanced tumor growth observed in transcription activator-like effector nuclease (TALEN)-mediated Ptch2 gene-edited mice models	(Veenstra et al., 2018; Byerly et al., 2022)
	GLI1	Hh transcription factor, increased nuclear localization during infection associated with delayed apoptosis.	RNA silencing attenuated stem-like properties in lung-adenocarcinoma cells and increased susceptibility to apoptosis.	(Po et al., 2017; Byerly et al., 2022)
	BCL-2	Anti-apoptotic protein, increased expression associated with delayed apoptosis during infection	High expression shown to confer growth advantage to human Epstein Barr Virus (EBV)-lymphoblastoid B cells.	(Warren et al., 2019; Byerly et al., 2022)
Hippo	YAP/TAZ	Hippo transcription factors, increased nuclear localization correlates with delayed host cell apoptosis during infection	Overexpression induces malignant transformation of human mammary epithelial cells	(Byerly et al., 2023; Luo et al., 2023)
	BCL-xL	Anti-apoptotic protein, increased expression associated with delayed apoptosis during infection	Promotes stemness and tumor progression in melanoma and glioblastomas	(Trisciuoglio et al., 2017; Byerly et al., 2023)
	GLUT1	Regulates glucose metabolism and BCL2 family of proteins (BCL-xL and BAX) to delay apoptosis during infection	Expression correlated with tumor proliferation in epithelial ovarian carcinoma	(Semaan et al., 2011; Byerly et al., 2023)
	APC	Degraded during infection to inhibit negative regulation of β-catenin and YAP.	C-terminal loss of function mutation disrupts tumor suppressive activity and promotes tumorigenesis in colorectal cancer.	(Zhang and Shay, 2017; Byerly et al., 2024)

The non-canonical Wnt/PCP pathway plays an important role in tumor development through its influence on cancer metastasis. Downstream signaling of the PCP pathway induces cytoskeletal rearrangement which facilitates cellular motility (Humphries and Mlodzik, 2018; Di Bartolomeo et al., 2023). In breast cancer, fibroblast-derived exosomes promote autocrine Wnt11/PCP signaling to enhance invasiveness. The invasive breast cancer cells displayed asymmetric localization of core PCP complexes like that found in development (Humphries and Mlodzik, 2018). In addition, there is a correlation between non-canonical Wnt, proinflammatory cytokines, and epithelial-mesenchymal transition (EMT). EMT induces metastasis in various cancer types and non-canonical Wnt signaling is commonly associated with EMT due to its role in cellular differentiation (Taciak et al., 2018). Likewise, proinflammatory cytokine interleukin (IL)-8 was found to induce EMT through Wnt signaling. Macrophages can limit cancer cell division through inhibition of canonical Wnt, but this increases non-canonical pathways. In cancer cells, increased non-canonical Wnt promotes differentiation, polarization, and separation from the tumor by EMT resulting in metastasis.

### Wnt signaling in *Ehrlichia*

**Figure 2:**
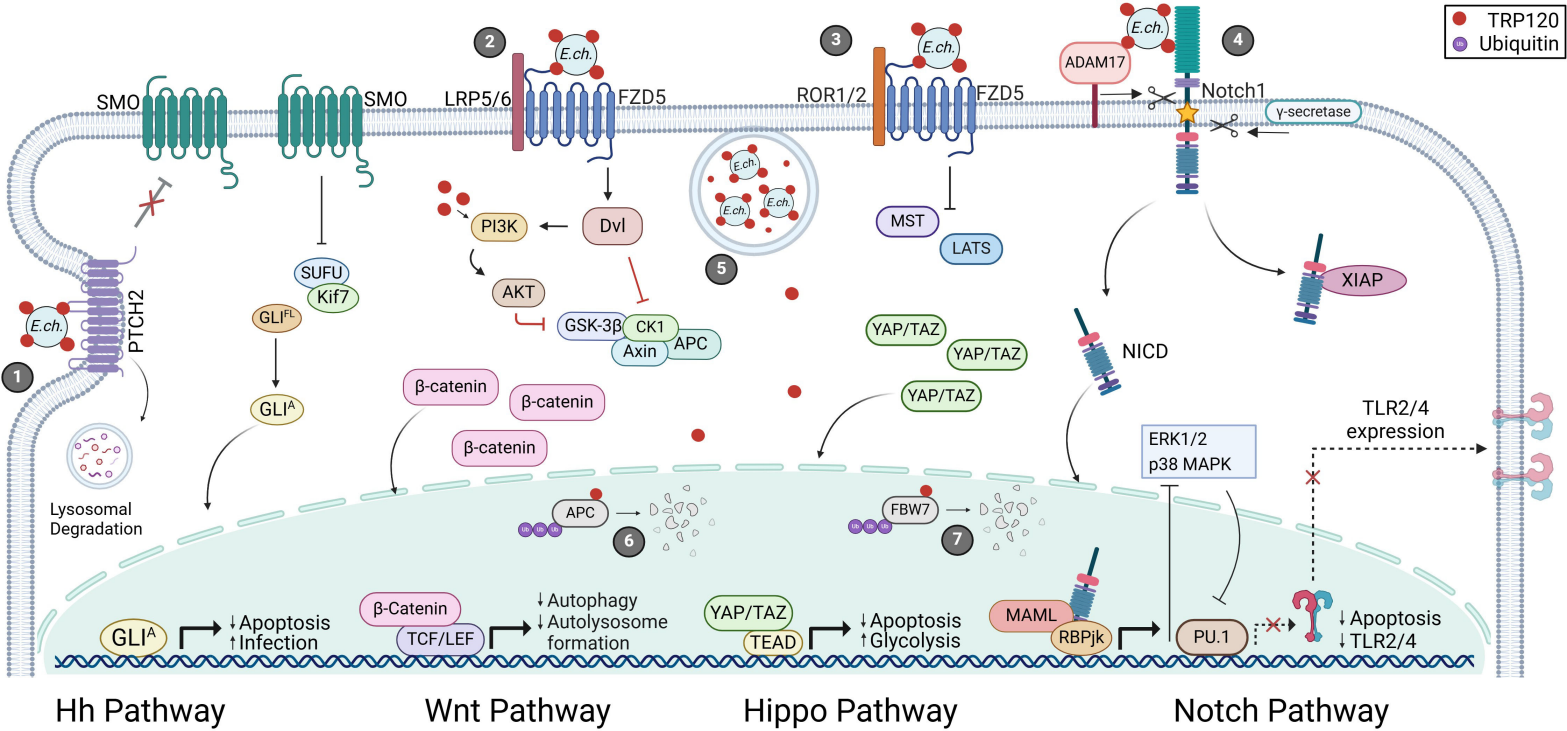
*E. chaffeensis* TRP120-mediated activation and functional outcomes of Hh, Wnt, Hippo and Notch signaling pathways. 1) Surface expressed TRP120 interacts with PTCH2 resulting in receptor internalization and lysosomal degradation. Active SMO accumulates and inhibits the SUFU protein complex releasing GLI to translocate to the nucleus and activate Hh target genes. 2) TRP120 binds FZD5 leading to the recruitment of co-receptor LRP5/6 and activation Wnt signaling activation. Active Dvl inhibits the β-catenin destruction complex (GSK3β, CK1, Axin, and APC) resulting in cytoplasmic accumulation of β-catenin and subsequent nuclear localization. 3) Additionally, TRP120 utilizes FZD5 to modulate Hippo signaling by inhibiting MST/LATS-mediated YAP/TAZ degradation. 4) Notch signaling is activated upon TRP120 interaction with Notch1 followed by receptor cleavage by ADAM17 and γ-secretase. Once cleaved, NICD stabilizes XIAP to inhibit apoptosis and translocates to the nucleus to regulate gene transcription. 5) *E. chaffeensis* enters host monocytes by receptor-mediated phagocytosis and reside in cytoplasmic vacuoles. TRP120 is secreted by the type-1 secretion system (T1SS) and translocates to the nucleus to mediate proteasomal degradation of 6) APC and 7) FBW7 to promote infection.

*E. chaffeensis* repurposes Wnt signaling to evade host immune responses and promote survival. Silencing of Wnt pathway components significantly reduces *E. chaffeensis* bacterial load, indicating the importance of Wnt signaling during infection. TRP120 contains a repetitive Wnt SLiM mimic (QDVASH) within the tandem repeat domain (TRD) (Rogan et al., 2021; Byerly et al., 2023). *E. chaffeensis* TRP120 Wnt SLiM mimic binds Fzd5 and induces nuclear translocation of β-catenin to modulate transcription of downstream Wnt target genes (**Figure 2**; **Table 1**) (Luo et al., 2016; Rogan et al., 2021). In this context, Wnt pathway activation results in cytoskeletal rearrangement and the induction of phagocytosis which contributes to ehrlichial host cell entry (Luo et al., 2016). Moreover, TRP120 has been shown to exploit the Wnt pathway to prevent autolysosome formation and allow *E. chaffeensis* to evade oxidative killing (Lina et al., 2017). Specifically, TRP120 binds to Wnt receptor and activates Dvl which subsequently activates the PI3K/AKT pathway and inhibits GSK3. PI3K/AKT activation inhibits negative regulator TSC2, which activates mTORC1. Activated mTOR phosphorylates TFEB, preventing nuclear translocation and subsequent upregulation of lysosomal target genes which prevents autolysosome formation (Lina et al., 2017). Additionally, canonical Wnt/β-catenin activation represses p62, an autophagy protein, as a mechanism for intracellular survival (Petherick et al., 2013). Interestingly, *E. chaffeensis* also activates the transcription factor nuclear factor of activated T-cells (NFAT) and initiates nuclear translocation of NFAT through the non-canonical Wnt/Ca2+ pathway. While knockdown of NFAT has been shown to significantly reduce *E. chaffeensis* bacterial load, the function of NFAT during infection has not been elucidated (**Table 1**) (Luo et al., 2016; Rogan et al., 2019).

## Notch pathway

Over a century ago, the Notch gene was discovered in *Drosophila melanogaster* (Zhou et al., 2022a). Genetic studies of *D. melanogaster* demonstrated that knockdown of the Notch gene was lethal (Zhou et al., 2022a). Since its discovery, Notch has been linked to developmental and cellular pathways including cell fate, proliferation, differentiation, adhesion, apoptosis, inflammation, and angiogenesis (Kumar et al., 2021). Furthermore, Notch dysregulation promotes cancer and infectious disease (Zhou et al., 2022a; Aster et al., 2017; Kumar et al., 2021). As a highly conserved and common target in disease, the Notch pathway is a prevalent area of research, as determining its interactions and roles in disease could lead to new therapeutic strategies (Aster et al., 2017; Kumar et al., 2021; Zhou et al., 2022a). Notch pathway activation is initiated by the interaction of Notch ligands (DLL1, -3, -4 and Jagged-1, -2) expressed on signal-sending cells and mature Notch receptors (Notch1, -2, -3 and -4) on signal-receiving cells that are glycosylated and cleaved in the Golgi by a Furin-like protease followed by receptor cleavage via ADAM17 and γ-secretase (Zhou et al., 2022a). The Notch intracellular domain (NICD) promotes transcription of Notch target gene families, including Hairy/Enhancer of Split (HES) and Hairy/Enhancer of Split related to YRPW motif (HEY), NF-κB, c-MYC, p21, through nuclear interactions with repressor CBF-1/suppressor of hairless/Lag1 (CSL/RBPJ) and transcriptional co-activator Mastermind-like protein (MAML) (**Figure 2**) (Capaccione and Pine, 2013; Zhou et al., 2022a). Notch, through nuclear and cytoplasmic interactions, modulates pathways associated with cell fate, proliferation, differentiation, adhesion, apoptosis, inflammation, and angiogenesis (Kumar et al., 2021).

### Notch signaling in cancer

As a key regulator of the immune response and cell fate, Notch associated genes are commonly mutated in several cancers. In breast cancer, genetic mutations upregulate Notch through gain-of-function mutations in *Notch1* (5-10%) and loss-of-function mutations in Numb (50%), a negative regulator of Notch (Stylianou et al., 2006; Aster et al., 2017). In T-cell acute lymphoblastic leukemia (T-ALL), loss-of-function mutations in F-Box and WD domain repeating containing 7 (FBW7) (20%) and gain-of-function mutation in *Notch1* (50-60%) also result in upregulated Notch signaling (Aster et al., 2017; Sanchez-Martin and Ferrando, 2017).

In both T-ALL and breast cancer, aberrant expression of Notch promotes cell proliferation and inhibits apoptosis (Stylianou et al., 2006; Palomero et al., 2007; Aster et al., 2017; Sanchez-Martin and Ferrando, 2017; Baker et al., 2018). Downstream effects include the inhibition of the JNK and p53 pathways resulting in decreased levels of pro-apoptotic factors, Puma and Noxa (Stylianou et al., 2006). Furthermore, Notch modulates the PI3K/AKT pathway through transcriptional downregulation of PTEN, a negative regulator of the PI3K/AKT pathway, in both T-ALL and breast cancer (Palomero et al., 2007; Baker et al., 2018).

Intriguingly, downregulation of PTEN and inhibition of FBW7 have both been associated with chemotherapeutic resistance. Both PTEN and FBW7 inhibition in cancer are associated with resistance to γ-secretase inhibitors (GSI) which are used to treat breast cancer and are known to downregulate Notch (Palomero et al., 2007; Thompson et al., 2007; Baker et al., 2018; Fan et al., 2022; Chen et al., 2023). In these cancers, upregulation of Notch promotes a tumorigenic environment through inhibition of apoptosis and regulation of cell growth and proliferation which encourages further resistance to chemotherapeutics.

### Notch signaling in *Ehrlichia*

During *E. chaffeensis* infection the Notch pathway is activated by TRP120 to inhibit apoptosis and promote infection. TRP120 promotes Notch activation through three mechanisms: direct activation of Notch, degradation of negative regulators, and transcriptional upregulation of Notch genes (Lina et al., 2016; Wang et al., 2020; Patterson et al., 2022, 2023). TRP120 contains an 11 amino acid SLiM (EDEIVSQPSSE) that mimics Notch ligands thereby activating Notch signaling during infection (Patterson et al., 2022). Moreover, TRP120 contains a HECT E3 ubiquitin ligase which ubiquitinates host FBW7, a negative regulator of NICD to maintain Notch activation (**Figure 2**) (Wang et al., 2020). FBW7 negatively regulates several oncoproteins (NICD, MCL-1, c-Jun, and c-MYC) through ubiquitination and subsequent proteasomal degradation (Wang et al., 2020). To further promote Notch activation, TRP120 binds the promoter regions of *Notch1* and *ADAM17* to promote transcription during infection (Lina et al., 2016). Upregulation of *Notch1* promotes generation of the Notch-1 receptor while *ADAM17* increases NICD S2 cleavage of the Notch receptors (Lina et al., 2016). *E. chaffeensis* activation of Notch inhibits PU.1, Toll-like receptor 2 and 4 (TLR2/4) expression through manipulation of ERK1/2 and p38 pathways (Lina et al., 2016). Further, X-linked inhibitor of apoptosis protein (XIAP) is sequestered and stabilized during infection due to increased cytoplasmic NICD (Patterson et al., 2023). Equally important, Notch activation leads to transcription of Notch target genes that modulate cell fate and proliferation to promote infection (**Table 1**) (Patterson et al., 2022).

## Hedgehog pathway

The Hedgehog (Hh) pathway is among a primary group of signaling pathways indispensable for embryonic development (Iriana et al., 2021; Zhou et al., 2022b). Hh signaling was first discovered in 1980 through mutagenesis screenings in *Drosophila* (Sari et al., 2018) and was found to be critical for embryogenesis, cell differentiation and tissue polarity (Iriana et al., 2021). Not surprisingly, aberrant Hh signaling results in developmental disorders and birth defects and has been shown to suppress host immune responses during tumorigenesis and pathogenic infections (Konstantinou et al., 2016; Smelkinson, 2017; Shi et al., 2018; Iriana et al., 2021). The Hh pathway is evolutionarily conserved among invertebrates and vertebrates, with pathway redundancy observed in the latter. In mammals, there are three Hh ligands, Sonic hedgehog (Shh), Dessert hedgehog (Dhh) and Indian hedgehog (Ihh) that bind to Patched1 (PTCH1) or Patched2 (PTCH2) receptors and activate glioma-associated oncogene (GLI) transcriptions factors, GLI-1, -2, or -3. Hh signaling has essential functions for cell-fate, pattern formation, proliferation and cell survival during development therefore dysregulated Hh signaling is associated with diseases such as Parkinsons’s, autism, epilepsy, osteoarthritis (OA), basal cell carcinoma (BCC), and pancreatic cancer (Li et al., 2021; Smith et al., 2022).

### Hedgehog signaling in cancer

Hh signaling is involved in numerous developmental processes, and thus is implicated in various genetic diseases, including cancer. The Hh signaling pathway is known to promote tumor formation via ligand-independent or ligand-dependent mechanisms. Hh ligand-independent cancers include basal cell carcinoma, medulloblastoma (MB) and pediatric brain tumors. Mechanisms required for ligand-independent cancers involve mutations in Hh pathway components that lead to constitutive activation of smoothened (SMO) and GLI and repression of PTCH and the suppressor of fused (SUFU) (Sari et al., 2018). Hh ligand-dependent cancers such as colorectal, ovarian, breast, prostate, pancreatic and liver cancers utilize either autocrine or paracrine signaling to promote tumorigenesis whereby endogenous ligands are copiously secreted, facilitating feed-forward pathway activation. Paracrine ligand-dependent Hh signaling requires endogenous ligands to bind stromal cell PTCH receptors thereby initiating the release of growth signals such as interleukin-6 (IL-6), vascular endothelial growth factor (VEGF), platelet derived growth factor (PDGF), bone morphogenetic protein (BMP), and insulin-like growth factor (IGF) to promote tumor progression (Sari et al., 2018).

### Hedgehog signaling in *Ehrlichia*

*E. chaffeensis* TRP120 engages the PTCH2 receptor through a repeated SLiM ligand mimic (NPEVLIKD) to activate Hh signaling. This activation results in nuclear translocation of GLI-1 in THP-1 cells and primary human monocytes (PHM) (**Figure 2**) (Byerly et al., 2022). Informational spectrum method (ISM) predicted the TRP120 Hh SLiM shares sequence and functional similarity with endogenous Hh ligands. This prediction was supported by protein interaction assays which demonstrated the tandem repeat domain of TRP120 co-localizes and directly interacts with the PTCH2 receptor. Furthermore, TRP120-mediated GLI-1 nuclear translocation resulted in upregulation of key target genes that were consistent with classical Hh ligands (**Table 1**) (Byerly et al., 2022).

During *E. chaffeensis* infection, Hh activation has been shown to significantly increase the expression of anti-apoptotic protein, BCL-2, thus preventing Bax-mediated cytochrome c release to maintain mitochondrial membrane integrity (**Table 1**) (Byerly et al., 2022). This ehrlichial survival strategy blocks intrinsic cell death signals and appropriates host cell nutrients for survival and dissemination. Further, knockdown of pathway components including GLI-1, PTCH2 and SMO decreases *E. chaffeensis* infection. In addition, THP-1 cell treatment with an antibody against the TRP120 Hh SLiM or treatment with a TRP120 Hh SLiM mutant prevented GLI-1 nuclear translocation and subsequent pathway activation. Moreover, *E. chaffeensis*-infected THP-1 cells showed decreased GLI-1 nuclear translocation and increased cell death after treatment with a Hh pathway inhibitor (Vismodegib/GDC-0449), suggesting that Hh signaling plays a significant role in *E. chaffeensis* infection by inhibiting apoptosis (Byerly et al., 2022). This study was the first to show *E. chaffeensis* TRP120 SLiM-mediated Hh activation, highlighting the necessity to understand the nuances of Hh signaling which will be fundamental in defining distinct mechanisms of pathway regulation in various diseases.

## Hippo pathway

Discovered in 2003, the Hippo signaling pathway is conserved in metazoans and essential in processes including regulation of organ size, organ homeostasis, and embryologic development (Liu et al., 2021; Xiao and Dong, 2021; Wang et al., 2021; Cox et al., 2018; Jiang et al., 2020). This pathway largely accomplishes its functions via control over cell survival and differentiation, and is generally influenced by signals including mechanical cues, stress, cell polarity, cell density, and soluble factors (Yu et al., 2012; Harvey et al., 2013; Misra and Irvine, 2018; Fu et al., 2022). Given the important cellular and developmental roles of Hippo signaling, it is not surprising that aberrant Hippo signaling results in many human diseases. Notably, the association of Hippo with cell proliferation, apoptosis, and survival is responsible for the high prevalence of abnormal Hippo signaling in malignancy.

### Hippo signaling in cancer

Although the role of Hippo signaling in cancer is context-dependent, the pathway is typically considered tumor suppressing. Thus, inactivation of Hippo signaling and downstream activation of Yes-associated protein 1 (YAP) and WW-domain-containing transcription regulator 1 (TAZ) is common in a variety of malignancies such as breast, gastric, renal, hepatic, and hematologic cancers (Xu et al., 2019; Kyriazoglou et al., 2021; Ma et al., 2021; Noorbakhsh et al., 2021; Song et al., 2022; Wu et al., 2022; Liu et al., 2023a; Messina et al., 2023; Li et al., 2024; Yang et al., 2024). Elevated activation of YAP/TAZ is implicated in tumor initiation, metastasis, and drug resistance through mechanisms including inhibition of apoptosis and reprogramming of metabolic pathways in tumor cells (Liu et al., 2021; Fu et al., 2022). Additionally, Hippo signaling engages in crosstalk with other pathways, such as Wnt signaling (discussed above), further mediating cancer cell survival and tumor progression (Jiang et al., 2020; Noorbakhsh et al., 2021; Li et al., 2019b).

When bound to transcriptional enhanced associated domain family of proteins (TEAD 1-4), YAP/TAZ may upregulate the expression of anti-apoptotic factors such as members of the BCL-2 and inhibitor of apoptosis protein (IAP) families (Cheng et al., 2022; Zhang et al., 2022). These proteins directly inhibit apoptosis by preventing apoptotic signals such as Bax/Bak-mediated mitochondrial outer membrane permeabilization or caspase cleavage, respectively (Cetraro et al., 2022; Czabotar and Garcia-Saez, 2023). Such mechanisms have been observed in malignancies including colorectal cancer and adenocarcinoma, amongst others (Zhang et al., 2015, 2022; Jin et al., 2021). YAP/TAZ activation can also contribute to metabolic reprograming in cancer cells, specifically in the upregulation of proteins that facilitate increased glucose uptake and glycolytic flux such as GLUT1-3, phosphofructokinase and others (Liu et al., 2021). These proteins not only support cancer cell growth by elevating energy acquisition but may also contribute to maintaining an anti-apoptotic profile by positive regulation of BCL-2 family proteins (Coloff et al., 2011; Liu et al., 2014; Fang et al., 2015; Lin and Xu, 2017; Li et al., 2019a). For example, GLUT1 upregulation is a consequence of YAP activation in both breast and gastric cancer and has been correlated with BCL-xL expression in colorectal and gastric cancer (Wincewicz et al., 2007; Lin and Xu, 2017; Li et al., 2019a). Interestingly, Hippo signaling also interacts with other signaling pathways and modulates cell survival through crosstalk (Jiang et al., 2020).

A key example of Hippo pathway crosstalk is that with the Wnt pathway. Activation of Hippo signaling impedes Wnt signaling, as phosphorylated, cytoplasmic YAP/TAZ sequesters β-catenin outside the nucleus, preventing its translocation and subsequent upregulation of Wnt target genes (Imajo et al., 2012). Therefore, it is unsurprising that some Wnt ligands, notably Wnt3a and Wnt5a, can inactivate Hippo signaling through Fzd5 to ensure successful β-catenin nuclear translocation (Park et al., 2015). The crosstalk between these pathways is particularly relevant in cancer, as Hippo inactivation and Wnt activation are common mechanisms of cancer cell survival (Jiang et al., 2020; Noorbakhsh et al., 2021; Li et al., 2019b; Andl and Zhang, 2017). YAP activation is essential for β-catenin function in a variety of cancers including melanoma, hepatoblastoma, colon cancer, and breast cancer, and a screen of 85 cancer cell lines determined that those driven by β-catenin were dependent on YAP (Rosenbluh et al., 2012; Tao et al., 2014; Liu et al., 2019; Quinn et al., 2021). Additionally, the latter study also concluded that β-catenin and YAP act as transcriptional coregulators, forming a complex that upregulates gene expression of anti-apoptotic proteins, survivin and BCL2L1 (Rosenbluh et al., 2012). Hippo and Wnt signaling are therefore inextricably linked and possess a great deal of influence over cell proliferation and survival. Intriguingly, co-option of the crosstalk between these two pathways has been observed during infection of host cells by *E. chaffeensis*, one of the few bacterial pathogens associated with Hippo signaling (Byerly et al., 2023).

### Hippo signaling in *Ehrlichia*

The role of Hippo signaling in bacterial infection is critically understudied for such a ubiquitous and influential signaling pathway. Interestingly, one of the best examples of Hippo pathway involvement in bacterial infection is *E. chaffeensis*, which is extensively demonstrated to inactivate Hippo through SLiM-icry. In fact, *E. chaffeensis* uses the same SLiM to both activate Wnt signaling and inactivate Hippo signaling, taking advantage of the crosstalk between these pathways. By inactivating Hippo signaling, *E. chaffeensis* ensures the efficacy of Wnt signaling activation while promoting YAP-mediated anti-apoptotic gene expression in host cells (**Figure 2**) (Byerly et al., 2023).

Motivated by the role of Wnt in *E. chaffeensis* pathology, and the existence of Wnt/Hippo crosstalk, YAP activation was investigated in a cell culture model of *E. chaffeensis* infection. In *E. chaffeensis-*infected cells, YAP is activated and translocates to the nucleus, where it upregulates a diverse panel of target genes. YAP activation during *E. chaffeensis* infection was attributed to the TRP120 Wnt SLiM, suggesting this sequence is responsible for Hippo inactivation in addition to activation of Wnt signaling. Furthermore, in Fzd5 knockout cells, YAP is not activated by *E. chaffeensis* or the TRP120 Wnt SLiM (Byerly et al., 2023). Finally, TRP120 ubiquitinates adenomatous polyposis coli (APC), a negative regulator of YAP and β-catenin, targeting it for degradation (**Figure 2**; **Table 1**) (Byerly et al., 2024). Taken together, these findings demonstrate that *E. chaffeensis* inactivates Hippo signaling through the same mechanism as Wnt signaling activation (Byerly et al., 2023).

As described above, inactivation of Hippo signaling may contribute to excessive cellular survival and metabolic reprogramming through YAP-mediated genetic regulation. Notably, this phenomenon is also observed in *E. chaffeensis*-infected cells. Hippo inactivation by *E. chaffeensis* is critical for pathogen survival as knockdown of YAP and TEAD family transcription factors significantly decreases infection (Byerly et al., 2023). Both *E. chaffeensis* and TRP120 Wnt SLiM significantly increase expression of GLUT1, while GLUT1 knockdown significantly decreases infection, suggesting this metabolic protein is crucial for maintaining infection (**Table 1**). Further investigation revealed that BCL-xL levels increase while Bax levels decrease in response to infection and the TRP120 Wnt SLiM, and this result is abrogated by treatment with Verteporfin, a YAP inhibitor (Liu-Chittenden et al., 2012; Byerly et al., 2023). Verteporfin also significantly decreases bacterial load and cell viability in infected cells, and significantly increases caspase activation, indicating an increase in apoptosis. Collectively, these results illustrate that *E. chaffeensis* inactivates Hippo signaling to engage the YAP-GLUT1-BCL-xL axis and establishes an anti-apoptotic profile in host cells, a mechanism like that observed in multiple cancers (Byerly et al., 2023).

## Conclusion and future perspectives

Cell death resistance and immune evasion are common survival strategies among various cancers and host-dependent pathogens. Given what is known about aberrant Wnt, Notch, Hh and Hippo signaling during oncogenesis and ehrlichial pathogenesis, it is important to understand pathway regulation in both contexts. Modulation of evolutionarily conserved embryonic pathways during *E. chaffeensis* infection is gaining attention as *E. chaffeensis* has proved a powerful model for investigating complex signal transduction pathways (Rogan et al., 2021; Byerly et al., 2022, 2023; Patterson et al., 2022; Pittner et al., 2023). Most importantly, *E. chaffeensis* has restructured our understanding of ligand binding requirements, challenging the long-accepted dogma of a tertiary ligand structure required for receptor interactions. It is important to understand SLiMs, not only as a biochemical phenomenon but as a means to broaden investigations of various diseases with the potential for SLiM-driven interactions, thereby improving the likelihood of SLiM-targeted therapies. The SLiM-mediated cellular interactions employed by *E. chaffeensis* have taught scientists the power of revisiting what was previously understood and has allowed appreciation of novel molecular strategies employed by a single bacterium.

SLiM-mediated pathway activation is not exclusive to *E. chaffeensis* (Van Roey et al., 2014; Elde and Malik, 2009; Pha et al., 2024; Giménez et al., 2024; Simonetti et al., 2023); however, unlike other infectious agents, *E. chaffeensis* has advanced our understanding of distinct mechanisms regulated by a single pathogen effector protein. Therefore, the use of *E. chaffeensis* to further study this phenomenon will undoubtably yield an even greater understanding of signaling pathways and their control over cell survival in cancer and intracellular infection. The balance of Wnt, Notch, Hh and Hippo signaling is crucial for stem cell development, cellular polarization, and differentiation. Consequently, aberrant signaling of these pathways have been heavily implicated in cancer and infectious diseases. Therapeutics targeting different components of these signaling pathways may be useful for treating cancer and infectious diseases. For example, the SMO inhibitor, Vismodegib, is an FDA approved cancer therapeutic used to treat basal cell carcinoma and while clinical trials are still ongoing to evaluate the efficacy of this drug in other tumors, the use of Vismodegib on *E. chaffeensis*-infected THP-1 cells to ameliorate cell death resistance associated with infection is a promising example of how understanding signaling mechanisms in cancer and pathogenic infections could improve standard clinical interventions for both diseases (Iriana et al., 2021; Byerly et al., 2022). Additionally, OMP-18R5 (vantictumab) interacts with Fzd5 to block Wnt activation and PKF115-584 inhibits the interaction between β-catenin and TCF/LEF, preventing gene activation (Tai et al., 2015). GSI inhibitors that prevent the cleavage and release of the NICD into the cytoplasm are promising therapeutics for aberrant Notch activation (Palomero et al., 2007; Baker et al., 2018). Furthermore, Verteporfin, a YAP inhibitor effective in preclinical studies of Hippo-implicated malignancies, was also demonstrated to significantly enhance apoptosis and decrease bacterial load in a cell culture model of *E. chaffeensis* infection (Wei and Li, 2020; Byerly et al., 2023).

Studies of *E. chaffeensis* have uncovered multiple SLiMs capable of modulating numerous signaling pathways, information useful as a tool in advancing general cellular and molecular approaches, design of pathway-modulating molecules, and detection of novel mechanisms in anomalous Wnt, Notch, Hh and Hippo signaling. There is growing evidence that several cancers are mediated by SLiMs including Burkitt’s lymphoma, prostate cancer, ovarian cancer and colorectal cancer (Vitari et al., 2011; Uyar et al., 2014; Kumar et al., 2024). The recognition of SLiM-mediated cancers has improved drug development efforts by exhibiting non-classical targets for therapeutics such as, Nutlins and Cilengitide. These drugs entered clinical trials as they have been shown to specifically target SLiM-mediated protein interactions in retinoblastoma, liposarcoma and glioblastoma (Uyar et al., 2014) signifying the possibility of targeting additional SLiM-mediated cancers in the near future. Not only is there opportunity to investigate SLiM-mediated pathway activation in cancers and other diseases, but researchers can now extend studies of *E. chaffeensis* as a tool to understand the signaling cascades reprogrammed in certain cancers, potentially improving therapeutic targets beyond globular protein-protein interactions. Due to the parallels of Wnt, Notch, Hh and Hippo signaling in cancer and *E. chaffeensis* infection, utilizing *E. chaffeensis* as a model to study aberrant signaling as it relates to cancer, intracellular pathogens, and production of novel therapeutics is essential.
